# Accreditation in the health sciences: a handbook for librarians

**DOI:** 10.29173/jchla29756

**Published:** 2024-08-01

**Authors:** Genevieve Forsyth

Schmick D. **Accreditation in the health sciences: a handbook for librarians**. 1st ed. London, UK: Rowman & Littlefield; 2023. Softcover: 220 p. ISBN: 978-1-5381-6557-7. Price: USD$80.00. Available from: https://rowman.com/ISBN/9781538165577/Accreditation-in-the-Health-Sciences-A-Handbook-for-Librarians

*Accreditation in the health sciences: a handbook for librarians* provides a thorough resource for librarians engaging in accreditation processes. There are few available comprehensive resources in this area, and this handbook is meant to fill this gap. It is edited by Darell Schmick, who is the inaugural Library Director at Noorda College of Osteopathic Medicine. Prior to this position, Schmick was the founding library director of the University of the Incarnate Word’s School of Osteopathic Medicine. Schmick led both of these institutions during their accreditation processes and based on his professional history, he has a wealth of knowledge in health sciences accreditation.

*Accreditation in the health sciences* is separated into twelve chapters along with an introduction and an epilogue, both authored by Schmick. Part I: Accreditation Fundamentals, includes discussions of different accrediting bodies, library preparation for the accreditation process, maintaining continuity through personnel changes, and a review of the first-time accreditation process. A highlight within Part I is Chapter one: accrediting bodies: history and context where the author, Mitzi R. Norris, provides much-needed context for the current constellation of accrediting bodies.

The next section is titled Part II: the accreditation process. These chapters focus upon the Magnet Recognition Program, Continuous Quality Improvement (CQI) processes, insights from accrediting officers, and best practices for library partnerships in the accreditation process, respectively. The Magnet Recognition Program recognizes institutions which “provid[e] nurses with a culture of empowerment that leads to improved patient outcomes to recruit high-quality nurses and demonstrate the ability to retain them.” Magnet is administered by the American Nurses Credentialing Centre (ANCC), and as per the authors, there are currently 547 organizations worldwide who have achieved Magnet designation. Much of the Virginia Commonwealth University library’s contribution to redesignation centred around supporting nurse engagement in Evidence-Based Practice (EBP). The authors Roy Brown and Alison Monpetit found that while EBP is highly valued in the Magnet core competencies, nurses at their institution reported barriers related to lack of time, knowledge and organizational support. Brown and Monpetit share that their library supported skill acquisition by collaborating with nurse educators and clinicians. Part of this work included librarians attending nursing meetings and building relationships with the nursing faculty. Librarians then aided nurses through providing training and support for those undergoing academic publishing projects. Monpetit and Brown state that investing librarian time and effort into EBP had a high return on investment for their organization. They argue that librarians should take a proactive approach to potential institutional designations, researching how they can aid in accreditation processes and serve as a connecting agent between educators, organizational leadership and clinicians.

In the next chapter, Charlotte M. Beyer provides a background on, and an argument for, Continuous Quality Improvement (CQI) within the library space. Beyer defines CQI as “constantly assessing internal processes while implementing improvements with the goal of providing high-quality outcomes.” Beyer emphasises that CQI is proactive in its approach to improving patient care and maximizing efficiency. Similar to EBP’s place in Magnet designation, evidence of organizational dedication to CQI has become a central tenet of the Liaison Committee on Medical Education (LCME)’s accreditation standards. All medical education programs leading to a Medical Doctor (MD) degree in the United States and Canada are accredited by the LCME. The discussion of benchmarking data collection and analysis within this chapter was also particularly useful. Data collection and analysis, while onerous, are the cornerstone of effective project management. As someone who has recently undertaken a major metrics project, Beyer’s “CQI Roadmap Using Benchmarking” would have been a great resource to consult beforehand. The questions Beyer poses within the roadmap establish and assess the ongoing collection of data ensures that it is being collected in an intentional, actionable way.

In these two chapters, the authors demonstrate that a proactive and collaborative approach from librarians and library staff is instrumental to a successful accreditation process. My takeaway from these chapters is that as librarians, our background in information literacy and research often aligns with the priorities of accrediting bodies. With institutional support, librarians can become subject matter experts on prioritized areas like EBP and CQI. We can then leverage this subject matter expertise and provide support to our organizations during the accreditation process. Doing so strengthens our organizations while also better integrating the library into the broader structure of the organization, which establishes connections between other departments and the library.

Part III: debrief: action items for follow up is the final part of the handbook, and it contains four chapters reviewing debriefing strategies, postmortems, strategies for continued partnerships, and assessment of information-seeking skills during curricular self-directed learning activities. Throughout the publication, all the accrediting bodies and all but one of the institutions mentioned are based in the United States. The one exception is the discussion of the Northern Ontario School of Medicine (NOSM) in Chapter 10: the post mortem: handling accreditation success and failure,” written by Joanne M. Mullenbach. In two pages, Mullenbach describes her experience at NOSM as the school sought accreditation from both the LCME and the Committee on Accreditation of Canadian Medical Schools (CACMS). Through the postmortem review of the accreditation journey, Mullenbach identifies that the library benefited from having a sufficient budget, and was therefore able to provide evidence of a functional, adequate library system to the LCME and CACMS. Mullenbach shares barriers faced during the project, including frequent senior administrative turnover and the technological challenges posed by being in a more remote, Northern environment in 2005. I was disappointed that her discussion of the topic was so brief and would have liked to read more about her experiences at NOSM.

While this book is overwhelmingly written about the accreditation process for American institutions, it is still a valuable resource for Canadian librarians. Firstly, there are currently no comprehensive Canadian counterparts to the handbook. Secondly, many accreditation programs mentioned in the book, including the Magnet Designation and the Council for Education in Public Health, do offer accreditation to Canadian organizations. Therefore, the discussion of library experience during these processes will be of interest. The topic coverage is quite broad, and the four parts of the book are organized in a sequential, logical manner. The book also offers a well-organized and extensive reference section, and a detailed index. The text is well-researched and contains many diverse case studies from institutions of different sizes, locations, and student demographics.

In the introduction to *Accreditation in the health sciences*, Schmick notes that this handbook was inspired by a gap he observed in resources for librarians working to support their institution’s accreditation processes. Having case studies and actionable recommendations in one volume is helpful for librarians and information professionals. I would also recommend this book to librarians researching how their teams can support transformative institutional changes and major projects. This book is priced at $80 USD and provides good value, given the comprehensive coverage of the subject matter. The focus on best practices and theory will ensure relevance as accreditation guidelines change. Overall, this handbook is a worthwhile resource for information about quality improvement processes, accreditation, and effective coordination for major projects.

